# Systematic study of single-cell isolation from musculoskeletal tissues for single-sell sequencing

**DOI:** 10.1186/s12860-022-00429-2

**Published:** 2022-07-26

**Authors:** Manman Gao, Peng Guo, Xizhe Liu, Penghui Zhang, Zhongyuan He, Liru Wen, Shaoyu Liu, Zhiyu Zhou, Weimin Zhu

**Affiliations:** 1 Department of Sport Medicine, Inst Translat Med, The First Affiliated Hospital of Shenzhen University, Shenzhen Second People’s Hospital, Shenzhen, China; 2 Department of Orthopaedic Surgery, The Seventh Affiliated Hospital, Sun Yat-Sen University, Shenzhen, China; 3 Guangdong Provincial Key Laboratory of Orthopedics and Traumatology, The First Affiliated Hospital of Sun Yat-Sen University, Guangzhou, China; 4 Shenzhen Key Laboratory of Anti-Aging and Regenerative Medicine, Department of Medical Cell Biology and Genetics, Health Sciences Center, Shenzhen University, Shenzhen, China

**Keywords:** Single-cell isolation protocol, Human musculoskeletal tissues, Efficiency, Quality

## Abstract

**Background:**

The single-cell platform provided revolutionary way to study cellular biology. Technologically, a sophistic protocol of isolating qualified single cells would be key to deliver to single-cell platform, which requires high cell viability, high cell yield and low content of cell aggregates or doublets. For musculoskeletal tissues, like bone, cartilage, nucleus pulposus and tendons, as well as their pathological state, which are tense and dense, it’s full of challenge to efficiently and rapidly prepare qualified single-cell suspension. Conventionally, enzymatic dissociation methods were wildly used but lack of quality control. In the present study, we designed the rapid cycling enzymatic processing method using tissue-specific enzyme cocktail to treat different human pathological musculoskeletal tissues, including degenerated nucleus pulposus (NP), ossifying posterior longitudinal ligament (OPLL) and knee articular cartilage (AC) with osteoarthritis aiming to rapidly and efficiently harvest qualified single-cell suspensions for single-cell RNA-sequencing (scRNA-seq).

**Results:**

We harvested highly qualified single-cell suspensions from NP and OPLL with sufficient cell numbers and high cell viability using the rapid cycling enzymatic processing method, which significantly increased the cell viability compared with the conventional long-time continuous digestion group (*P <* 0.05). Bioanalyzer trace showed expected cDNA size distribution of the scRNA-seq library and a clear separation of cellular barcodes from background partitions were verified by the barcode-rank plot after sequencing. T-SNE visualization revealed highly heterogeneous cell subsets in NP and OPLL. Unfortunately, we failed to obtain eligible samples from articular cartilage due to low cell viability and excessive cell aggregates and doublets.

**Conclusions:**

In conclusion, using the rapid cycling enzymatic processing method, we provided thorough protocols for preparing single-cell suspensions from human musculoskeletal tissues, which was timesaving, efficient and protective to cell viability. The strategy would greatly guarantee the cell heterogeneity, which is critical for scRNA-seq data analysis. The protocol to treat human OA articular cartilage should be further improved.

**Supplementary Information:**

The online version contains supplementary material available at 10.1186/s12860-022-00429-2.

## Background

Since establishment of the scientific discipline of cellular biology and development of the cell theory in 1839 [1], the researchers’ endeavor to enrich the field has never been halted. The milestone theory states that the cell is the basic unit of structure and organization of living organisms. In any multicellular organism, cell heterogeneity is one of the most prominent characteristics, which is critical to its peculiar function and fate. Similarly, living tissues are composed of diversiform cell types and each cell type is generally incorporated with genetically heterogeneous cell-subpopulations [2]. Due to this complexity, the conventional analyses of cellular biology using bulk of tissues or cells are apparently limited. The emerging field of single-cell techniques provides promising tools to characterize the cellular biology at the resolution of individual cells [3, 4].

Single-cell genomic technologies, coincided with transformative new methods to profile genetic, epigenetic, spatial, proteomic and lineage information, have revolutionized the research methodology for exploring biological systems [5]. Technologically, the workflow mainly includes four parts: tissue procurement and single-cell isolation, Single-Cell Sequencing library construction and quality control, library sequencing, and data analysis. As rapid development and maturation of single-cell platform, high quality input of single-cell suspension has become the prerequisite factor for ideal output datasets [6]. For nonadherent cells like peripheral blood mononuclear cells, it’s much easier to gain single-cell suspension. While for adherent and even solid tissues, it’s a challenge to dissociate cells from extracellular matrix without injuring cell viability. Especially for musculoskeletal system, including bones, muscles, joints, cartilage, ligaments, tendons and bursae, tissues of which are dense and tense.

Commonly, protocols for tissue dissociation mainly include mechanical dissection, enzymatic breakdown and combinatorial methods. In any event, little damage and high efficiency are the baseline for viable single-cell isolation. Although, Magnetic-activated cell sorting (MACS) and fluorescence-activated cell sorting (FACS) could be used to discard the dead cells to meet the requirements of single-cell platform, high fraction of dead cells per se would compromise heterogeneity. Therefore, high cell viability should be defended during sample preparation. Traditionally, various enzymatic digestion protocols were employed for cell isolation from bone, cartilage, nucleus pulposus, and tendons, but generally neglected the quality control. In 2019, Ninib Baryawno et al. reported the Single-Cell RNA Sequencing (scRNA-seq) results of mice bone and bone marrow [7]. The enzymatic solution used in their study was 1 mg/mL STEMxyme1 (Worthington, Ref# LS004106) and 1 mg/mL Dispase II (ThermoFisher Scientific, Ref# 17105041) supplemented in Media 199 with 2% FBS (shown as the S-D solution in this paper). Taking advantage of the digestion medium, they rapidly obtained a highly viable and multifarious single-cell mixture with mesenchymal stem/stromal cells, osteo-lineage cells, chondrocytes, fibroblasts, endothelial cells, and pericytes, suggesting a broad-spectrum sensitivity and high efficiency to various tissues but moderate effect to cell viability.

However, human musculoskeletal tissues are much trickier than the ones from mouse. Combined with the S-D solution and conventional methods, we designed the rapid cycling enzymatic processing method using tissue-specific enzyme cocktail to treat human NP, OPLL and AC. The specific enzyme cocktail showed high efficiency in dissociation single cells. Nevertheless, compared with the regular once-through digestion method, the rapid cycling enzymatic processing method showed higher cell viability. High digestion efficiency and cell viability could greatly guarantee the cell heterogeneity to be interpreted. Taking advantage of the protocols, we efficiently prepared qualified single-cell suspensions from NP and OPLL for single-cell platform. However, the single-cell suspensions from articular cartilage were hardly qualified using no matter rapid cycling or once-through long-time digestion method due to excessive cell aggregates and doublets or damaged cell viability, respectively, suggesting more protocols should be tested. In conclusion, we provided thorough protocols for preparing single-cell suspensions rapidly and efficiently from human musculoskeletal tissues using tissue-specific enzyme cocktail and the rapid cycling enzymatic processing method, which would contribute to exploring the cell heterogeneity.

## Results

### Validation of the single-cell isolation protocol for human degenerated NP

**Fig. 1:**
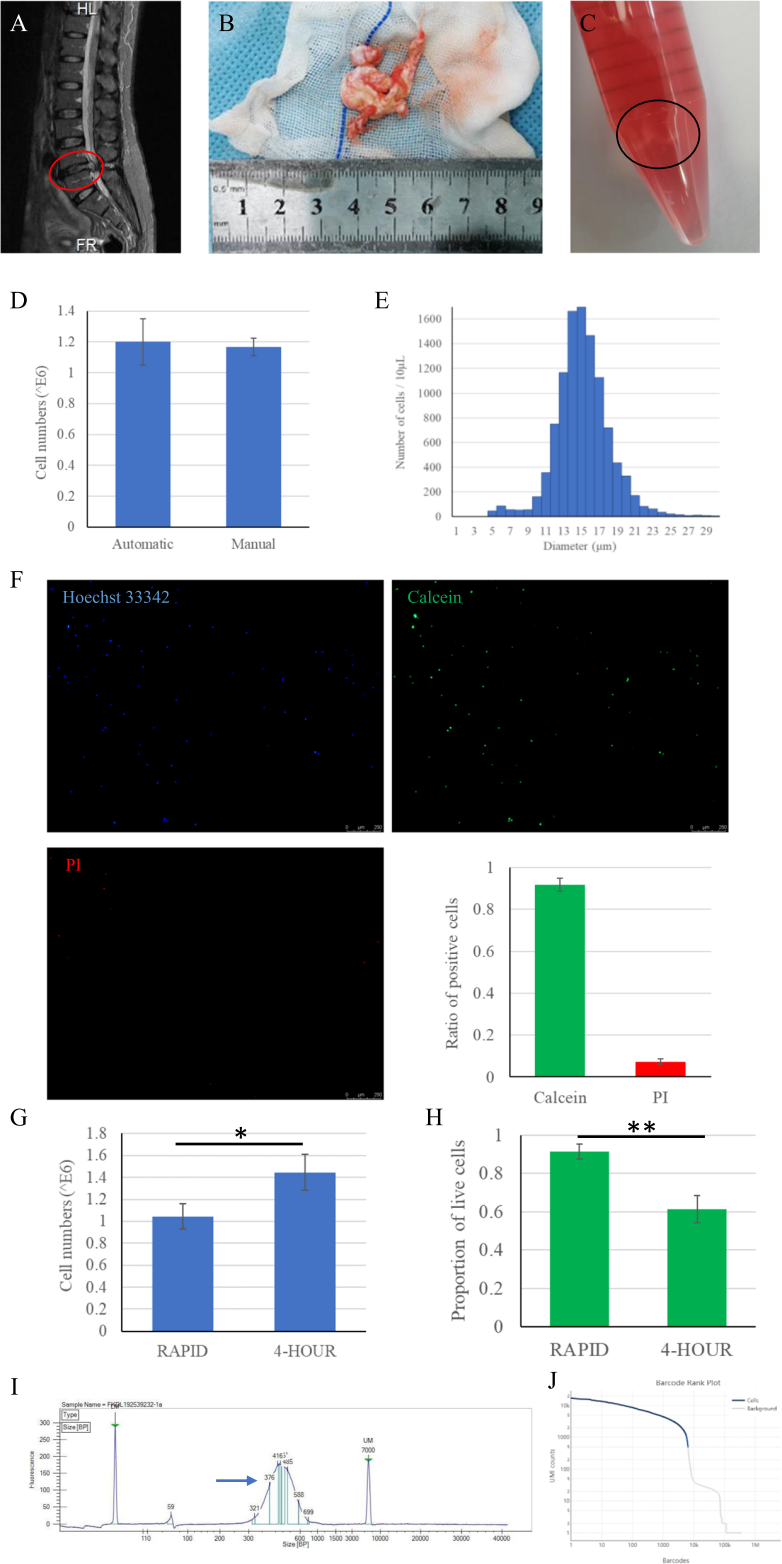
Isolation of single cells from degenerated nucleus pulposus (NP) and Single-Cell RNA Sequencing (scRNA-seq). A. Lumber disc degeneration was shown on T2-weighted (T2-WI) Magnetic Resonance Imaging (MRI). The red circle marked the degenerated disc. B. Gross appearance of the degenerated NP obtained from surgery. C. Gross appearance of the cell suspension after 3–5 cycles of enzymatic treatment. Some white floccule residuals were labeled by black circle. D. Total cell numbers were counted automatically and manually, respectively. E. The isolated cell diameter distribution histogram. G. Histogram showing the cell output harvested via the rapid cycling enzymatic processing method (RAPID) or 4-h once-through digestion method (4-HOUR). H. Histogram showing proportion of live cells in each group. I. The live/dead immunofluorescence staining and quantitative analysis. J. Bioanalyzer trace of the scRNA-seq library showed the size distribution between 300 and 600 bp (blue arrow). H. The barcode-rank plot displayed gene expression counts after sequencing

Human degenerated NP tissues (Fig. 1B) were harvested during surgery from patients suffering degenerative disc disease (Fig. 1A). After 3–5 cycles of digestion, the NP fragments were markedly diminished and only few white floccules were remnant (Fig. 1C). The suspension became viscous, which was probably resulted from increased concentration of proteoglycans disaggregated from extracellular matrix [8, 9]. Using the rapid cycling enzymatic processing method, we harvested sufficient cells for following experiments, which were 1.20 × 10^6^ ± 0.15 × 10^6^ cells counted automatically and 1.15 × 10^6^ ± 0.06 × 10^6^ cells counted manually (Fig. 1D). According to the cell diameter distribution, cells with diameter ≧ 20 μm occupied 6.6 ± 0.6% (Fig. 1E). Live/dead assays showed 91.7 ± 3.2% of cells were Calcein^+^ and 7.3 ± 1.2% were PI^+^. There were few observed cell aggregates or doublets (Fig. 1F), which corresponded with the cell diameter distribution. The cell viability was significantly damaged when using the 4-h once-through digestion method, which indicated a decreased proportion of live cells to 61.3 ± 7.0% (*P* < 0.01) (Fig. 1G), although the cell output was increased (1.04 × 10^6^ ± 0.11 × 10^6^ and 1.44 × 10^6^ ± 0.16 × 10^6^, *P* < 0.05) (Fig. 1H). Three qualified samples harvested through the rapid cycling enzymatic processing method were sent to scRNA-seq library construction and sequencing. Quality of the library was validated by Agilent Bioanalyzer 2100 (Fig. 1I). After sequencing, the barcode rank plot exhibited a steep slope (Fig. 1J), suggesting a clear separation of cellular barcodes from background partitions. T-SNE visualization showed high cell heterogeneity in all samples compared with the published studies [10–14] (Table 1, Supplementary Fig. 1). Above all, using the NP-specific enzyme cocktail and rapid cycling enzymatic processing method, highly qualified single cell suspensions could be efficiently prepared for sc-RNA seq platform.

**Table 1:** Summary of single-cell isolation protocols for nucleus pulposus in reported studies using scRNA-seq technique

Reference	Classification of IVD	Species	Treatment	Dead cell removal	Profiling cellular types
Gan Y, et al. [10]	Pfirrmann grade I	Human	TrypLE Express for 30 min, 0.2% pronase (Sigma-Aldrich, USA) for 60 min, and 0.012% collagenase II (Sigma-Aldrich, USA) for 2 ~ 4 h, sequentially	Yes	Chondrocyte, Notochord, Nucleus pulposus progenitor cells, Stromal cell, Pericyte, Endothelial cell and blood cell
Fernandes LM, et al. [11]	Thompson grade 1 or 2	Human	Pronase for 1 h and then collagenase P overnight	No Culture cells for scRNA-seq	Cultured nucleus pulposus cells
Calió M, et al. [12]		Bovine	0.19% Pronase for 1 h and then 260 IU/mg Collagenase II overnight	No	Nucleus pulposus cells, uncharacterized cell subset, monocyte derived dendritic cells
Zhang Y, et al. [13]	Pfirrmann grade I-IV	Human	GEXSCOPETM Tissue Dissociation Solution for 15 min. (Included in the GEXSCOPE® Single Cell RNA library Kit)	No	Chondrocytes, macrophages and endothelial cells
Ji Tu, et al. [14]	Pfirrmann grade II-V	Human	500 U/mL collagenase I, 150 U/mL collagenase II, 50 U/mL collagenase IV, 0.1 mg/ml hyaluronidase and 30 U/mL DNaseI for 95 min	Yes	Neucleus pulposus cells, natural killer cells, monocytes, and T cells

### Validation of the single-cell isolation protocol for human ossifying posterior longitudinal ligaments

**Fig. 2:**
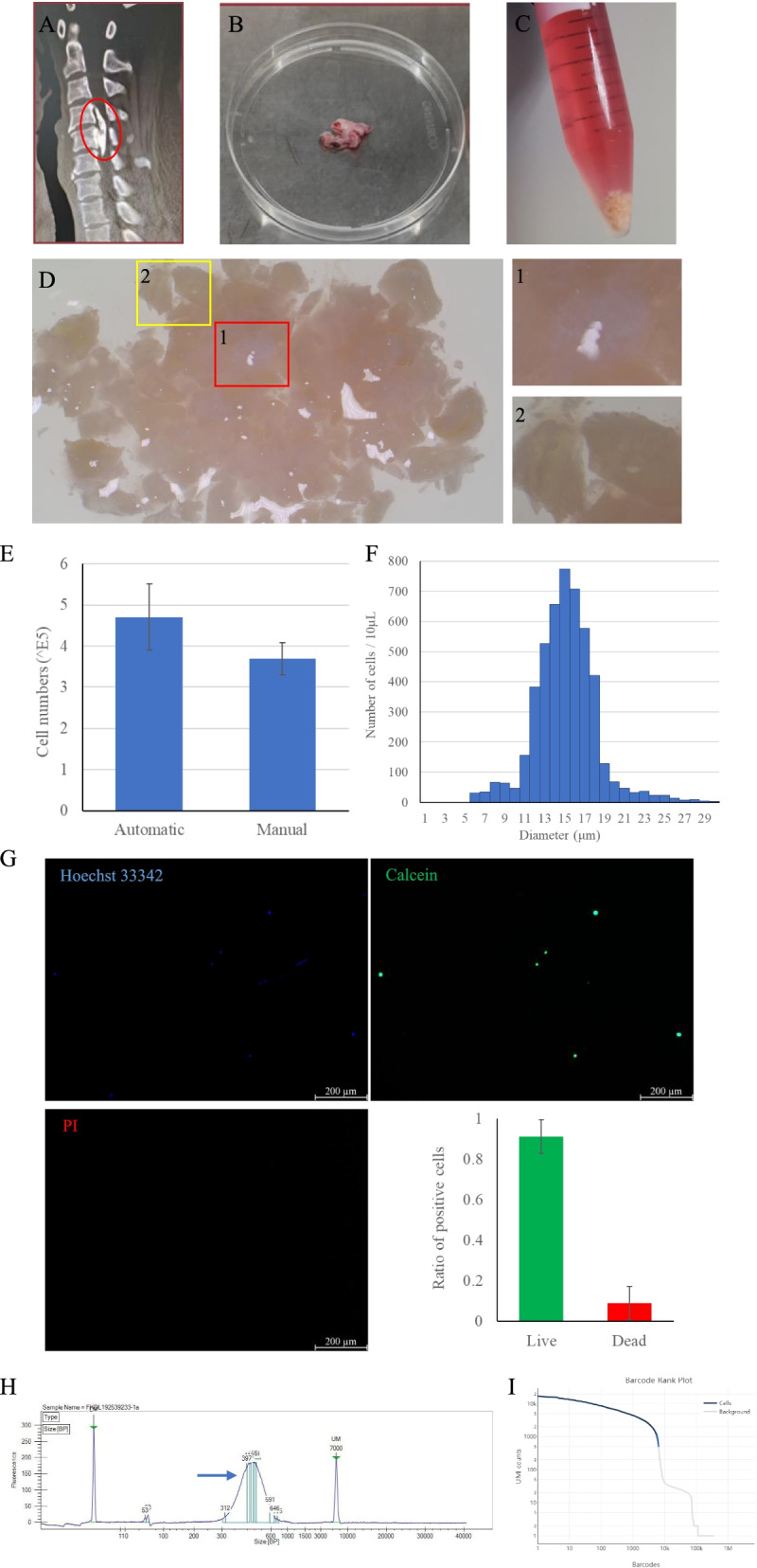
Isolation of single cells from ossifying posterior longitudinal ligament and scRNA-seq. **A**. Ossification of posterior longitudinal ligament (OPLL) was shown on the three-dimensional reconstruction of computerized tomography (CT) image (red circle). **B**. Gross appearance of the ossifying posterior longitudinal ligament harvested during surgery. **C**. Gross appearance of the cell suspension and residuals after 3–5 cycles of enzymatic digestion. **D**. Stereo microscope images showed the residuals. The red box enclosed some white soft viscous residuals magnified in the image labeled 1. And the yellow box marked part of the bone fragments magnified in the image labeled 2. **E**. Total cell numbers were counted automatically and manually, respectively. **F**. The isolated cell diameter distribution histogram. **G**. The live/dead immunofluorescence staining and quantitative analysis. **H**. Bioanalyzer trace of the scRNA-seq library showed the size distribution between 300 and 600 bp (blue arrow). **I**. The barcode-rank plot displayed gene expression counts after sequencing

Ossifying posterior longitudinal ligaments were reported composed with bone-like tissues, cartilage-like tissues, none-ossifying ligaments, bone marrow, etc. To improve efficiency in dissociation the intricately composed tissues, collagenase type I, frequently used in digestion of bone and tendons, was combined with the S-D solution in the present study. Ossifying posterior longitudinal ligaments were obtained from patients with ossification of posterior longitudinal ligament (OPLL) during surgery (Fig. 2A, B). After 3–5 cycles of digestion, the left tissues were mainly bone-like fragments (Fig. 2C, D). Total cell number counted automatically was 4.71 × 10^5^ ± 0.81 × 10^5^ cells and manually was 3.70 × 10^5^ ± 0.39 × 10^5^ cells (Fig. 2E). The outcome of considerable remnant bone fragments suggested that bone was more difficult to be processed than soft tissues in short time [15, 16]. Cells with diameter ≧ 20 μm occupied 6.3 ± 1% of total cells according to the cell diameter distribution (Fig. 2F) and there were no observed cell aggregates or doublets (Fig. 2G). High cell viability was verified by the live/dead assays, with 91.1 ± 8.4% of cells Calcein^+^ and 8.9 ± 8.4% of cells PI^+^ (Fig. 2G). We then carried out scRNA-seq library construction and sequencing for two of the qualified samples. Bioanalyzer trace was shown to validate the library quality (Fig. 2H). And barcode rank plot suggested a clear separation of cellular barcodes from background partitions (Fig. 2I), indicating good quality of scRNA-seq library. T-SNE visualization showed high cell heterogeneity in both samples (Supplementary Fig. 2), confirming the digestion efficiency and protection to cell viability. In summary, using the above enzyme cocktail and rapid cycling enzymatic processing method, qualified single-cell suspensions could be harvested from the intricately composed ossifying posterior longitudinal ligaments.

### Failed single-cell preparation from human knee articular cartilage with OA

**Fig. 3:**
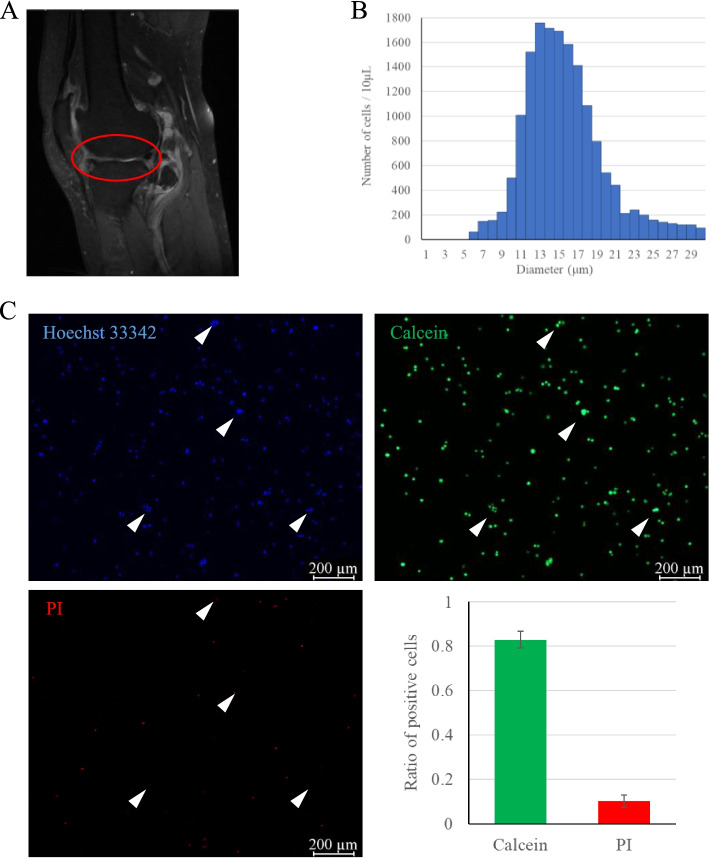
Rapid isolation of single cells from knee articular cartilage with osteoarthritis (OA) and the quality control. **A**. OA was characterized on T2-weighted imaging (T2-WI). The affected region was circled in red. **B**. The isolated cell diameter distribution histogram. **C**. The live/dead immunofluorescence staining and quantitative analysis. White arrowheads highlighted the cell aggregates, which showed Hoechst 33342^+^, Calcein^+^ and PI^−^

**Fig. 4:**
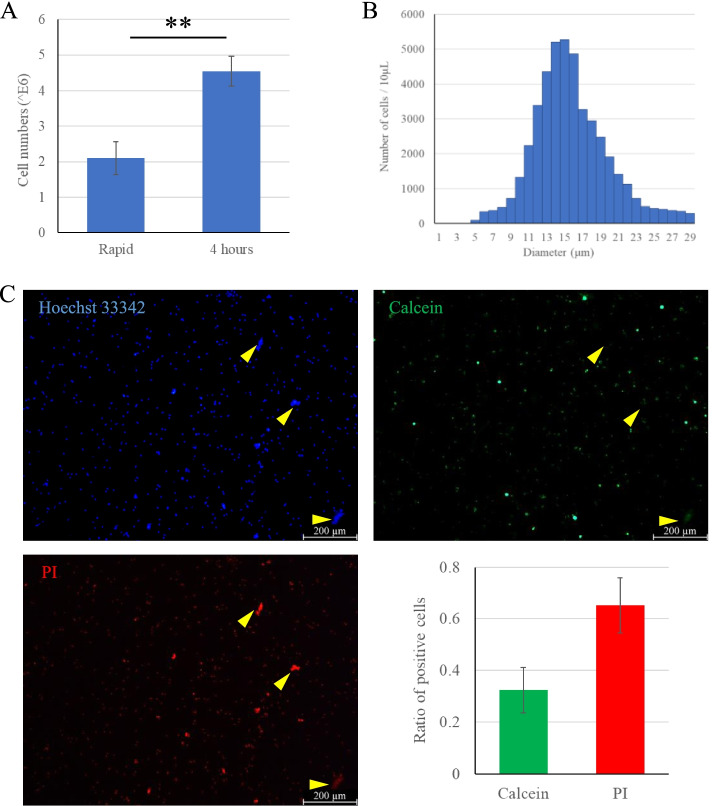
Prolonged isolation of single cells from knee articular cartilage with OA and the quality control. **A**. The total cell numbers counted automatically in both rapid and prolonged enzymatic treatment groups. Prolonged and continuous digestion would significantly increase cell yield (*P* < 0.01). **B**. The isolated cell diameter distribution histogram. **C**. The live/dead immunofluorescence staining and quantitative analysis. Yellow arrowheads highlighted the cell aggregates, which showed Hoechst 33342^+^, Calcein^−^ and PI^+^

Collagenase type II was wildly used in dissociation articular cartilage, which was added with the S-D solution in this study. After digestion, filtration and wash, lots of cell aggregates were observed under microscope and only 83.0 ± 3.8% of cells were viable (Fig. 3C). Cells with diameter ≧ 20 μm occupied 12.6 ± 3.1% (Fig. 3B). Fortunately, most of the cell aggregates were viable (Fig. 3C), suggesting an insufficient digestive efficiency. According to Quanbo Ji’s research, we prolonged the enzymatic treatment to increase the efficiency. The cell yield was significantly increased compared with the rapid cycling treatment group (Fig. 4A). However, the ratio of cells with diameter ≧ 20 μm was unexpectedly increased to 22.6 ± 2.2% (*P* < 0.05) (Fig. 4B). Live/dead assays showed 65.2 ± 10.6% of cells were dead (*P* < 0.05) (Fig. 4C), including most of the cell aggregates.

## Discussion

Development of scRNA-seq technique contributes a big step for cellular biology. Preparation of qualified single-cell suspension is a precondition for the platform. It’s full of challenge to offer eligible samples from dense and tense musculoskeletal tissues, like bone, cartilage, nucleus pulposus, tendons, etc. In the present study, we aimed to provide standard and specific protocols for preparing qualified single-cell suspensions from human degenerated nucleus pulposus, ossifying posterior longitudinal ligaments and knee articular cartilage with OA. As a result, the protocols for the former two types of tissues could be applied to output qualified samples for following scRNA-seq. However, dissociated cell suspensions from knee articular cartilage were unqualified due to either excessive aggregates and doubles or severely damaged cell viability, which needed more efforts in the future.

Nucleus pulposus, the inner part of intervertebral disc, performs critical biomechanical functions [17–19]. Accumulated evidences supported that the nucleus pulposus degenerated at early stage of degenerative disc disease [20–22], which is a common cause of disability and results in a huge socioeconomic impact world widely [23]. The etiology as well as pathology is complex and multifactorial [18, 24]. Although the underlying pathology has been approached from a variety of ways [18], a systematically study to get a panorama is still missing. The single-cell platform provided a revolutionary way to solve this problem, but there was no related report till now. Therefore, to harvest qualified single-cell suspension would be the first step. In previous studies, NP was usually dissociated via various enzymatic methods and the treatment duration was from 30 min to overnight, which was summarized detailly in Juliana’s paper [25]. As is well known, long time and intense enzymatic treatment would increase cell yield while damage cell viability, vice versa. And the lack of systematic quality control made it hard to be directly used in single-cell isolation.

Pronase, a mixture of several nonspecific endo- and exoproteases, has been wildly used in isolating cells from NP [26]. It was reported that Pronase treatment would shorten the required digestion time [25]. Based on these researches, we combined Pronase with the S-D solution for single-cell isolation from NP in this study and obtained highly qualified single-cell suspensions possessing abundant cell yield, high cell viability, and few cell aggregates or doublets. In conclusion, the enzymatic treatment protocol for human NP was validated for single-cell platform.

The posterior longitudinal ligament runs entire length of the spine. Ossification of the posterior longitudinal ligament (OPLL), defined as an ectopic ossification (calcification), would lead to paralysis of extremities and disturbances of motility [27]. Up to now, the osteo-lineage derivation, cell atlas transformation and molecular regulation have never been elucidated, which retarded the development of etiological treatment [27–29]. Single-cell platform, taking advantage of horizontal cell atlas and vertical trajectory analysis, would broaden the horizons in this field. Normal posterior longitudinal ligament is fibrous connective tissue. However, the ossifying posterior longitudinal ligament is a mixture of ligament, bone and probably cartilage as a result of endochondral ossification process [30, 31]. It’s full of challenge to ascertain one digestion medium to dissociate mixed tissues. Collagenase type I, frequently used in digestion of bone and tendons, was combined with the S-D solution in the present study. Moderate cell yield, high cell viability and low content of cell aggregates or doublets were realized using the present protocol. Although scRNA-seq was conducted, the cell heterogeneity should be inspected carefully by data analysis. In particular, the quantity and variety of bone derived cells should be noted in consideration of lots of bone-like tissue remnants. More experiments will be carried out to raise the cell yield but not compromise on cell viability.

Articular cartilage is specialized connective tissue of diarthrodial joints and functions as a load-bearing, low-friction, and wear-resistant surface to facilitate joint movement [32]. As it is lack of self-regenerative ability, articular cartilage is one of the most common degenerative tissues. Osteoarthritis (OA) is the primary joint disorder and a major cause of disability, affecting 303 million people globally in 2017 [33, 34]. The etiology, pathology and regenerative therapy have been wildly studied [35–38]. Also utilizing the single-cell platform, there were two nice reports that systematically uncovered the cell atlas and transcriptional regulation mechanisms of OA [39, 40]. As for other related studies, several reports showed the single-cell profiling results of developing limbs, including growth plate cartilage as well as articular cartilage [41–44]. The single-cell isolation methods used in the above studies were summarized in Table 2. In Quanbo Ji’s research, the articular cartilage was obtained from 10 patients but only 1600 cells were sequenced, suggesting a too moderate digestive efficiency. In Fiorella Carla Grandi’s study, the primary chondrocytes were delivered to culture and treatment in vitro before scRNA-seq, the quality control of isolated primary single cells was not reported. And in other researches, samples were obtained from mice but not human.

**Table 2:** Summary of single-cell isolation protocols for cartilage in reported studies using scRNA-seq technique

Reference	Tissue	Species	Treatment	Number of profiling cells
Quanbo Ji, et al. [34]	Cartilage from 10 knee OA patients	Human	0.25% Trypsin–EDTA for 30 min and then 0.2% type II collagenase (Sigma-Aldrich) for 4 h	1600
Fiorella Carla Grandi, et al. [35]	OA samples from patients undergoing total joint replacement	Human	Collagenase II and IV (2.5 mg/ml each; Worthington Biochem) overnight	Not reported
Junxiang Li, et al. [36]	The distal cartilage structure at postnatal day 7	Mouse	0.2% collagenase II for 2 h	217
Vikram Sunkara, et al. [37]	Femur of 12 weeks old mice	Mouse	10 mg/ml Collagenase II (Nordmark) supplemented for 4 h	7133
Koji Mizuhashi, et al. [38]	Femur growth plate cells at postnatal day 2	Mouse	Liberase TM (Sigma/Roche) for unclear time	18,000
Natalie H Kelly, et al. [39]	Hindlimb bud of embryonic mice	Mouse	Collagenase Type II (Worthington-biochem, Lakewood, NJ) and pronase (EMD Millipore, Billerica, MA) in 15 min increments with agitation for up to 1 h	9466

Collagenase type II was wildly used in dissociation articular cartilage, which was added with the S-D solution in this study. Using the rapid protocol, excessive live cell aggregates suggested insufficient enzymatic efficiency but longer duration of treatment significantly damaged cell viability with plenty of dead cell aggregates. Different from insufficient digestion, the dead cells could be aggregated into various sizes by released DNA [45]. DNase could be used to degrade DNA and reduce dead cell aggregation [46], and FACS or MACS could be used to remove the dead cells [47, 48]. However, the potential loss of cell heterogeneity might mislead the data analysis. So longer enzymatic duration was not a better choice.

For hard dissociated tissues, single-nucleus RNA-sequencing (snRNA-seq) is an appealing alternative choice. In this method, a mild and quick nuclear dissociation protocol is used to isolate and sequence RNA within the nucleus, which minimizes technical issues that can arise from common dissociation protocols [49, 50]. More detailed explorations will be performed in the future.

## Conclusions

Preparing highly qualified single cell suspensions is the precondition for single-cell sequencing technologies and indiscriminatingly capture of all kinds of cell subsets is pivotal to interpretate the cellular biological networks. In the present study, a thorough protocol for preparing single-cell suspensions from human musculoskeletal tissues is provided, which was timesaving, efficient and protective to cell viability. Although the protocol to treat human OA articular cartilage should be further improved, the rapid cycling enzymatic processing method would greatly guarantee the cell heterogeneity.

## Methods

### Preparation of the enzymatic solution

Media 199 (ThermoFisher Scientific, Ref# 12350039) supplemented with 2% Fetal Bovine Serum (FBS, ThermoFisher Scientific, Ref# 10082147) was used as basic medium. STEMxyme1 (Worthington, Ref# LS004106), Dispase II (ThermoFisher Scientific, Ref# 17105041), Pronase (Roche, Ref# 10165921001), Collagenase type I (ThermoFisher Scientific, Ref# 17100017) and Collagenase type II (ThermoFisher Scientific, Ref# 17101015) were respectively dissolved in 1 × phosphate-buffered saline (PBS) buffer to a concentration of 10 mg/mL. Filter the solution with a 0.22-μm membrane filter and store at -20℃. The enzymatic solution for NP was prepared by adding STEMxyme1, Dispase II and Pronase solutions into the basic medium to the concentration of 1 mg/mL, 1 mg/mL and 2 mg/mL, respectively. For ossifying posterior longitudinal ligaments, STEMxyme1, Dispase II and Collagenase type I solutions were added into the basic medium to the same concentration of 1 mg/mL. Similarly, STEMxyme1, Dispase II and Collagenase type II solutions were added into the basic medium to the same concentration of 1 mg/mL, which was used in single-cell dissociation from OA articular cartilage. All enzymatic solutions must be freshly prepared each time.

### Human samples

The degenerated degree of intervertebral disc was assessed according to the modified Pfirrmann grading system using preoperative magnetic resonance imaging (MRI). In total of six intervertebral disc samples classified as grade 3 degeneration were harvested during discectomy. Three of them were processed in the rapid cycling enzymatic processing method and sent to sc-RNA seq and the others were divided into two groups to compare different processing methods. The OPLL diagnosis was confirmed by X-ray, computed tomography (CT) and MRI. Three samples were obtained during anterior cervical decompression surgery. The severity of knee OA was evaluated based on Kellgren-Lawrence (K-L) scoring system. Three diseased articular cartilage specimens were acquired from patients undergoing knee arthroplasty surgery and each of the specimen was divided into 2 groups to compare different processing methods.

**Fig. 5:**
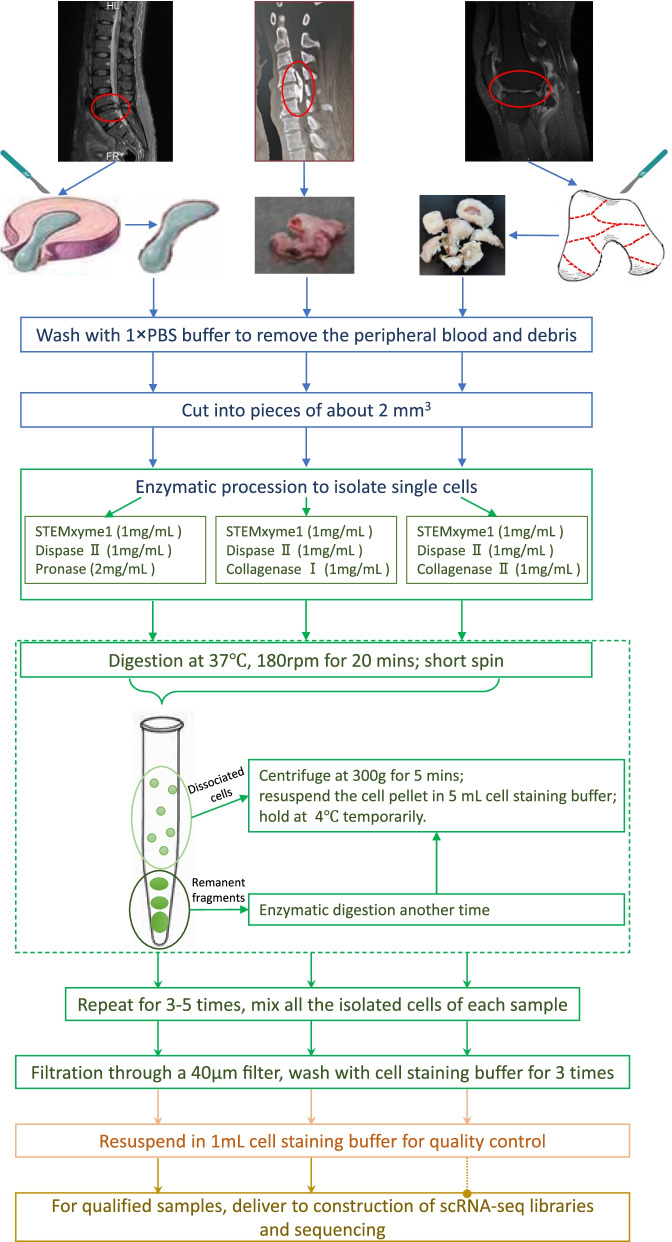
Schematic illustration of single cell isolation protocols from different musculoskeletal tissues

All human samples were hold in normal saline in ice-water bath as soon as resected during surgery and delivered to the following treatment in one hour. For intervertebral disc samples, annulus fibrosus was carefully removed to remain the nucleus pulposus. And the diseased articular cartilage was shaved from the underlying bone and cut into several pieces. All specimens were washed 3–5 times using ice-cold 1 × PBS buffer to remove the peripheral blood and debris. Schematic illustration of the protocols was exhibited in Fig. 5.

### Isolation of single cells in rapid cycling enzymatic processing method

Firstly, the specimens were added 0.3 mL of the enzymatic solution on the surface and minced into pieces of about 2 mm^3^. Then each 100 mg specimen was added with 10 mL enzymatic solution with agitation (180 rpm) at 37℃ for 20 min. Transiently centrifuge at 300 g for 10 secs, transfer the supernatant cell suspension into a new tube and keep the residual tissue fragments at 4℃ temporarily. Next, centrifuge the cell suspension at 300 g for 5 min. The supernatant enzymatic solution was added back into the tissue fragments for second digestion and the collected cell pellet was dispersed in 5 mL cell staining buffer (Biolegend, Ref# 420201). Keep the dispersed cells at 4℃ temporarily. Repeat the above operations for 3–5 times, and collect all cells after filtration through a 40 μm filter into a collection tube. In this method, timely collection of dispersed cells would protect them from excessive digestion, and cyclic digestion would raise the cell yield. Wash with cell staining buffer for 3 times and resuspend the collected cells in 1 mL cell staining buffer for following operations. The residual tissue fragments were observed under stereo microscope (Olympus, SZX7).

### Isolation of single cells in once-through digestion method

The specimens were added 0.3 mL of the enzymatic solution on the surface and minced into pieces of about 2 mm3. Then each 300 mg specimen was added with 10 mL enzymatic solution with agitation (180 rpm) at 37℃ for 4 h. Collect all cells after filtration through a 40 μm filter into a collection tube. Wash with cell staining buffer for 3 times and resuspend the collected cells in 1 mL cell staining buffer for following operations. The residual tissue fragments were observed under stereo microscope (Olympus, SZX7).

### Cell counting and quality control

Cell counting was performed both automatically (JIMBIO, JIMBIO CL) and manually. For automatic cell counting, 10μL cell suspension was added into the sample injector for detection. Each sample was repeatedly tested for 3 times. After automatic cell counting, pipette 100μL cell suspension for the following manual cell counting and dilute the cell suspension if the cell concentration was over 200 cells/μL. The Neubauer chambers were employed (BRAND, Ref# BR717810). Firstly, put the glass cover on the chamber central area. Then pipette 10μL cell suspension and slowly expel the liquid into the counting chamber avoiding air bubbles. Using the 10X objective, count the total number of cells found in 4 large corner squares under the microscope and calculate the cell counts using the formula below:

$$Cells/\mu L=\frac{Counted cells}{Counted surface\left({mm}^{2}\right)\cdot Chamber depth\left(mm\right)\cdot Dilution}$$

Counted surface(mm2) = 1 mm2 × 4 (area of four large corner squares), Chamber depth = 0.1 mm

The live/dead assays were detected using triple-staining with Hoechst 33342 (Invitrogen, Ref# R37605), calcein AM (Invitrogen, Ref# C1430) and propidium iodide (PI, Invitrogen, Ref# P3566). The fluorescent stains were added in 100μL cell suspension and incubated for 15 min at room temperature in dark. Hoechst 33342 was added 5μL (a ready-to-use reagent kit) into the cell suspension, and the final work concentration of calcein AM was 5 μM and PI was 0.5 μg/mL. At the same time, the blank control and negative control were prepared. For the blank control, same doses of dyes were added into 100μL cell staining buffer with no cells to determine the level of the background signal. And for the negative control, cells were pre-treated with 0.2% Triton X-100 (Sigma-Aldrich, Ref# T8787) for 15 min at 37° C, washed with 1 × PBS buffer for 3 times, resuspended in cell staining buffer and added same doses of dyes for incubation. The negative control could help estimate the background signal of calcein from dead cells. After incubation, pipette 10μL cell suspension into the Neubauer counting chamber and observe under fluorescence microscope.

All the tests, including cell counting and live/dead assays, were repeated for 3 times for each sample.

### Construction of scRNA-seq libraries and quality control

The construction of scRNA-seq libraries was strictly performed according to the instructions. Briefly, qualified samples were identified as containing more than 85% viable cells, less than 10% doublets and no cell aggregates. Two of the qualified samples were delivered to scRNA-seq library construction and sequencing. Approximately 10,000 cells per sample were loaded onto 10X Genomics Chromium 3’ Single Cell Gene Expression Solution v3 micro fluidics chip (10X Genomics, Ref# PN-2000060) to generate Gel Beads-in-emulsion (GEM) with barcodes. After break of the GEMs, reverse transcription was applied to generate the first-strand cDNA with purification using Silane magnetic beads. And then, barcoded and full-length cDNA was amplified via PCR to generate sufficient mass for library construction. Enzymatic fragmentation and size selection were used to optimize the cDNA amplicon size. After sequential add with P5, P7, a sample index and TruSeq Read 2, the libraries were constructed. The quality of the scRNA-seq libraries was analyzed by Agilent Bioanalyzer 2100 before Next-generation sequencing.

### Statistical analysis

The statistical analysis was performed by SPSS 16.0 statistical software. Student's t-test was utilized after performance of Shapiro-Wilks Normality Test. Quantitative data was presented as mean ± SE. *P*-value of less than 0.05 was considered to be statistically significant.

## Supplementary Information

**Additional file 1**: **SupplementaryFigure 1.** T-SNE plots showing cells distributionfrom each sample of human degenerative nucleus pulposus. Different cell subsetswere indicated with different numbers. **Supplementary Figure 2**.T-SNE plots showing cells distribution from each sample of human ossifiedposterior longitudinal ligament. Different cell subsets were indicated with different numbers.

**Additional file 2:** 

## Data Availability

The datasets used and/or analyzed during the current study are available from the corresponding author on reasonable request.

## Abbreviations

scRNA-seq: Single-cell RNA-sequencing
snRNA-seq: Single-nucleus RNA-sequencing
the S-D solution: 1 Mg/mL STEMxyme1 (Worthington, Ref# LS004106) and 1 mg/mL Dispase II (ThermoFisher Scientific, Ref# 17105041) supplemented in Media 199 with 2% FBS
MACS: Magnetic-activated cell sorting
FACS: Fluorescence-activated cell sorting
PI: Propidium iodide
NP: Nucleus pulposus
OA: Osteoarthritis
OPLL: Ossification of the posterior longitudinal ligament
GEM: Gel Beads-in-emulsion
