# Supplementary material for: Systematic study of single-cell isolation from musculoskeletal tissues for single-sell sequencing
Source: BMC Mol Cell Biol. 2022 Jul 26;23:32. doi: 10.1186/s12860-022-00429-2 (PMC9327421; doi:10.1186/s12860-022-00429-2)
Supplement: Supplementary file 1 — Additional file 1: SupplementaryFigure 1. T-SNE plots showing cells distributionfrom each sample of human degenerative nucleus pulposus. Different cell subsetswere indicated with different numbers. Supplementary Figure 2.T-SNE plots showing cells distribution from each sample of human ossifiedposterior longitudinal ligament. Different cell subsets were indicated with different numbers. [file 12860_2022_429_MOESM1_ESM.pptx]

## Slide 1
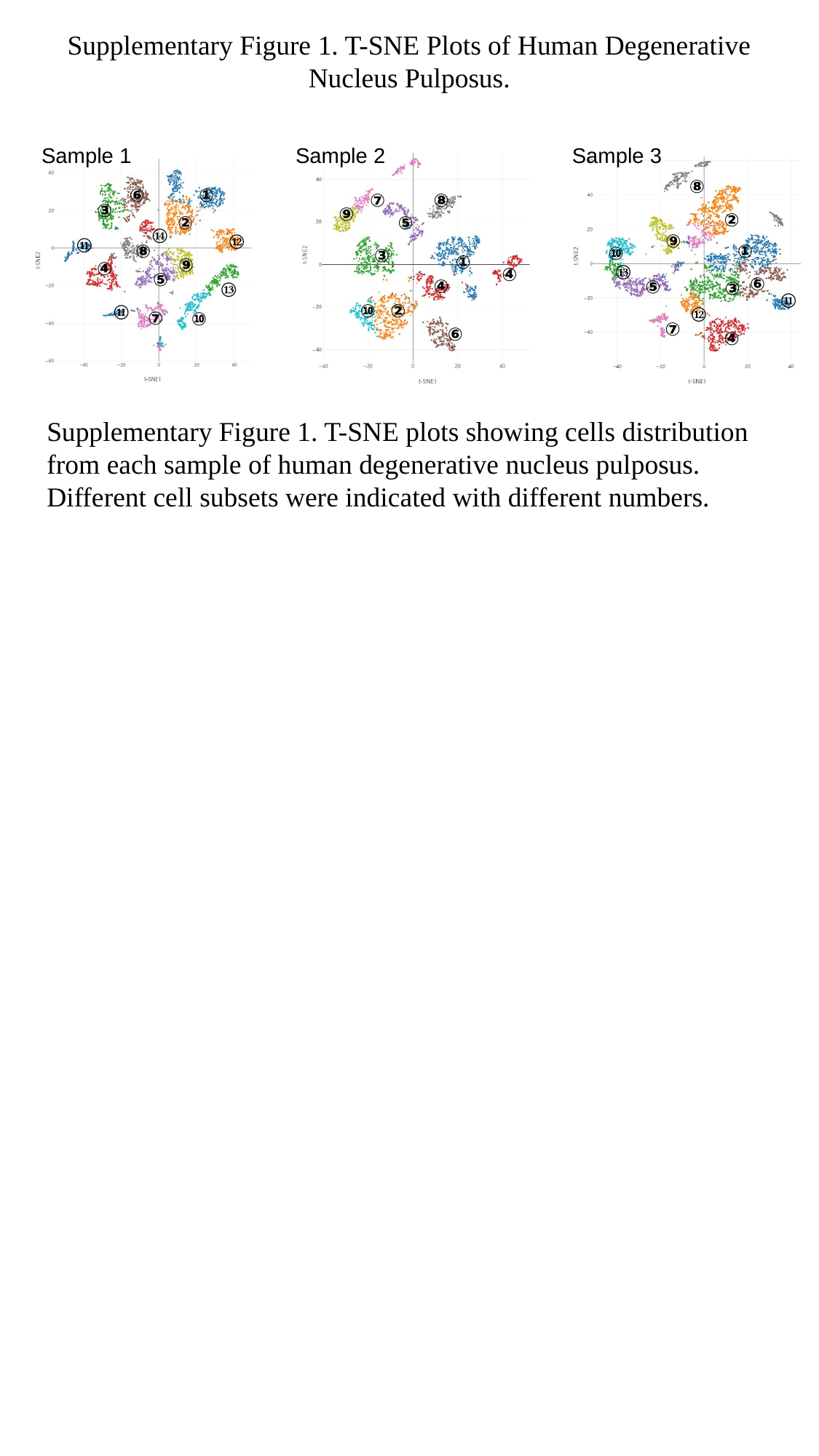

Supplementary Figure 1. T-SNE Plots of Human Degenerative Nucleus Pulposus.
Sample 1
⑥
①
③
②
⑭
⑫
⑪
⑧
⑨
④
⑤
⑬
⑪
⑦
⑩
Sample 2
⑦
⑧
⑨
⑤
③
①
④
④
⑩
②
⑥
Sample 3
⑧
②
⑨
①
⑩
⑬
⑥
⑤
③
⑪
⑫
⑦
④
Supplementary Figure 1. T-SNE plots showing cells distribution from each sample of human degenerative nucleus pulposus. Different cell subsets were indicated with different numbers.

## Slide 2
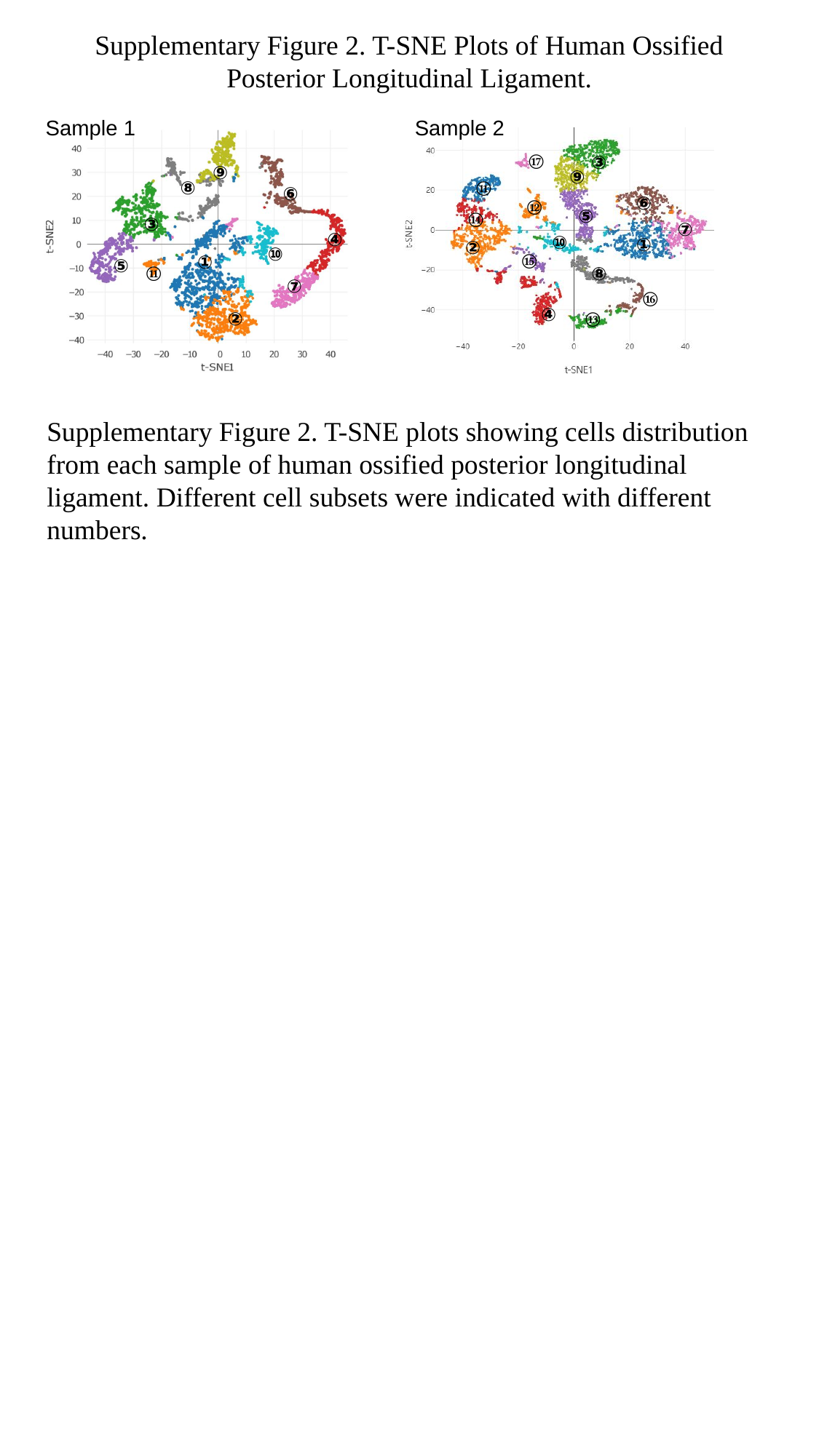

Supplementary Figure 2. T-SNE Plots of Human Ossified Posterior Longitudinal Ligament.
Sample 1
⑨
⑧
⑥
③
④
⑩
①
⑤
⑪
⑦
②
Sample 2
⑰
③
⑨
⑪
⑥
⑫
⑤
⑭
⑦
⑩
①
②
⑮
⑧
⑯
④
⑬
Supplementary Figure 2. T-SNE plots showing cells distribution from each sample of human ossified posterior longitudinal ligament. Different cell subsets were indicated with different numbers.
