# Supplementary material for: Systematic study of single-cell isolation from musculoskeletal tissues for single-sell sequencing
Source: BMC Mol Cell Biol. 2022 Jul 26;23:32. doi: 10.1186/s12860-022-00429-2 (PMC9327421; doi:10.1186/s12860-022-00429-2)
Supplement: Supplementary file 2 — Additional file 2: [file 12860_2022_429_MOESM2_ESM.docx]

October 10, 2021

The editors

BMC Molecular and Cell Biology

Dear Editors,

We greatly appreciate the valuable comments and suggestions from the editors and the reviewers to our previous submission with the manuscript entitled ‘Systematic Study of Single-Cell Isolation from Musculoskeletal Tissues for Single-Cell Sequencing’ (CEBI-D-20-00266R2). We have modified the manuscript accordingly upon the comments. Enclosed please find the point-by-point response and the revised manuscript.

We hope that this revision is acceptable. Thank you for your time and consideration to our work.

Yours sincerely,

Weimin Zhu, Zhiyu Zhou

On behalf of all authors

Correspondence to:

Weimin Zhu, Department of Sport Medicine, Inst Translat Med, The First Affiliated Hospital of Shenzhen University, Shenzhen Second People's Hospital, Shenzhen, China

Telephone: +86-0755-83791860, Email: [szhzwm@email.szu.edu.cn](mailto:szhzwm@email.szu.edu.cn)

Zhiyu Zhou, Department of Orthopaedic Surgery, The Seventh Affiliated Hospital, Sun Yat-sen University, Shenzhen, China

Telephone: +86-0755-81206999, Email: zhouzhy23@mail.sysu.edu.cn

**Revision Highlight**

The author’s responses to the editor’s comments are in red, for example, ABC; The author’s responses to the reviewer#1’s comments are in green, for example, CDF; The author’s responses to the reviewer #2’s comments are in blue, for example, GHI. The author’s responses to the reviewer #3’s comments are in orange, for example, JKL. Please find the following changes in this revision.

**Editor Comments:**

The reviewers recognize that the revised manuscript is much improved on the first submission to the journal. However, there are outstanding concerns regarding the statistical analysis and comparison to previous methods. If additional replicates are available we would you encourage you to include these in the analysis to strengthen the conclusions. At a minimum would ask that you include the statistical analysis request by Reviewer 2 and discuss the advantages of this method compared to others methods.

Authors: Thanks for your suggestions. Firstly, we added 3 more NP samples to compared the **rapid cycling enzymatic processing method** in the present study with the previously regularly used **once-through digestion method**. Results showed that using the first method significantly increased the cell viability which is key for cell heterogeneity interpretation. Moreover, 3 NP samples and 2 OPLL samples were sent to sc-RNA seq. T-SNE plots shown in supplementary figures indicated repeatable high cell heterogeneity. The manuscript was thoroughly modified according to your suggestions and requirements. Above all, the rapid cycling enzymatic processing method put forward in this study would be conducive to improving cell viability in live single cell isolation experiments, including sc-RNA seq, flow cytometry and others.

Reviewer reports:

Reviewer 1: When asked about statistical analysis, the authors cited that "Actually, we have more samples successfully processed and finished the single cell RNA-Seq, and the interpretation results will be published later..."; and "We did not compare more methods to processing these tissues but would rather provide the most practicable one. So, there was few statistical analyses to compare different processing methods.", so the authors may include pilot data, for example to demonstrate the feasibility of your approach. However, your pilot studies and results should be send to "scientific report" or "brief communication". For a manuscript, I suggest increasing the amount of sample sufficient for statistical analysis.

Authors: Thanks for your suggestions. We appreciated your valuable comments concerning the statistical analysis. To improve the manuscript and strengthen the conclusion, we added 3 more NP samples to compare the rapid cycling enzymatic processing method with the regularly used once-through digestion method, which showed that using the first method would significantly increase the cell viability. Moreover, 3 NP samples and 2 OPLL samples were sent to sc-RNA seq. T-SNE plots shown in supplementary figures indicated repeatable high cell heterogeneity.

The information in the M&M, the authors should provide a figure showing the gating strategy for all used panels (Live/Dead kit - Calcein vs PI).

Authors: Thanks for your suggestions. In cell viability assays, the staining results were observed under fluorescence microscope. Blank control added with no cells and negative control added with cells pre-treated with 0.2% Triton X-100 were prepared to help determine the background signal. The results were not detected by flow cytometry.

Standardize the graphics used. The information in figure 2F, 3G, 4C, and 5C, for example.

Authors: Thanks for your suggestions. We have standardized the fluorescent staining results.

Reviewer 2: The authors have answered properly my questions. They also have modified the manuscript improving their quality. I recommend their publication.

Authors: Thanks for your positive comments and careful review.

Reviewer 3: The authors addressed my concerns and questions.

However, I would recommend to add the preliminary t-sne plots (in the review document) to the manuscript supplementary figure. i understand that the authors want to follow-up on them in a second study and in that case, just the t-sne plots don't take anything away.

Authors: Thanks for your positive comments and careful review. We have added the T-SNE plots presenting cellular heterogeneity to support the conclusion.
